# Association of Dengue Virus and *Leptospira* Co-Infections with Malaria Severity 

**DOI:** 10.3201/eid2608.191214

**Published:** 2020-08

**Authors:** Rajendra Mandage, Charandeep Kaur, Atreyi Pramanik, Vinod Kumar, Parul Kodan, Adarsh Singh, Sounak Saha, Shivam Pandey, Naveet Wig, Ravindra Mohan Pandey, Manish Soneja, Pragyan Acharya

**Affiliations:** All India Institute of Medical Sciences, New Delhi, India (R. Mandage, C. Kaur, A. Pramanik, P. Kodan, S. Pandey, N. Wig, R.M. Pandey, M. Soneja, P. Acharya);; Nehru Shatabdi Chikitsalaya, Singrauli, India (V. Kumar);; Indian Institute of Technology, Kharagpur, West Bengal, India (A. Singh, S. Saha)

**Keywords:** parasitology, parasites, microbiology, malaria, plasmodium, severe malaria, mild malaria, dengue, Leptospira, scrub typhus, Orientia tsutsugamushi, bacteria, viruses, acute febrile illness, co-infection

## Abstract

*Plasmodium* infections are co-endemic with infections caused by other agents of acute febrile illnesses, such as dengue virus (DENV), chikungunya virus, *Leptospira* spp., and *Orientia tsutsugamushi*. However, co-infections may influence disease severity, treatment outcomes, and development of drug resistance. When we analyzed cases of acute febrile illness at the All India Institute of Medical Sciences, New Delhi, India, from July 2017 through September 2018, we found that most patients with malaria harbored co-infections (*Plasmodium* mixed species and other pathogens). DENV was the most common malaria co-infection (44% of total infections). DENV serotype 4 was associated with mild malaria, and *Leptospira* was associated with severe malaria. We also found the presence of *P. knowlesi* in our study population. Therefore, in areas with a large number of severe malaria cases, diagnostic screening for all 4 DENV serotypes, *Leptospira*, and all *Plasmodium* species should be performed.

In tropical countries, including India, acute febrile illnesses (AFIs) constitute a group of infections with similar manifestations, such as fever, malaise, body aches, chills, hepatic and renal dysfunction, and central nervous system effects. The causative agents of AFI can be bacterial (e.g., *Orientia tsutsugamushi, Leptospira*, and *Salmonella enterica* serovar Typhi), parasitic (protozoans of the apicomplexa family), or viral (e.g., dengue virus [DENV], chikungunya virus [CHIKV], influenza A[H1N1] virus) (*1*–*4*). Distinguishing between the causative agents of AFIs can be difficult. In tropical climates, several AFI pathogens, such as malaria parasites, DENV, and CHIKV, occur in the same areas and during the same seasons (*5*), making it possible that >1 pathogen can infect the same person. Indeed, recent retrospective analyses based on persons hospitalized with an AFI have uncovered malaria co-infections with dengue, chikungunya, and leptospirosis in different populations across the world (*6*–*14*).

Despite the increasing realization that co-infections may contribute to the course and outcome of malaria, only a few studies have investigated the prevalence and nature of co-infections (*14*–*20*), which limits our ability to manage and understand AFIs, as follows. First, we do not know the spectrum of infections that a person with an AFI may harbor, leading to inadequate drug therapy. Treatment strategies based on diagnosis of a single pathogen may lead to inadvertent exposure of the undetected pathogen to antimicrobial agents, thereby contributing to generation of antimicrobial-resistant species. Second, lack of adequate data on co-infections in clinical and field settings can misdirect the field of drug and vaccine development. Pathogens such as malaria parasites, DENV, and *Orientia* spp. have host immune-modulatory effects (*21*). Therefore, co-infections can aid or antagonize each other in terms of evading host immune responses. These interactions may have major effects on immune responses to vaccine candidates and need to be known during design of effective vaccination strategies (*22*). Third, we do not know how interactions of co-infecting pathogens lead to diverse disease outcomes affecting organ function and ultimately mortality. In India, the prevalence of malaria parasites, DENV, and CHIKV resembles the global prevalence and co-endemicity of these pathogens (*5*). Malaria infections in India are reportedly caused by *Plasmodium falciparum*, *P. vivax, P. ovale*, and *P. malariae* (*23*). Several studies have also reported the occurrence of *P. vivax* severe malaria in India as well as in Southeast Asia and South America (*23*–*25*). Our objective with this study was to define the spectrum of co-infections in patients with an AFI associated with malaria admitted to the All India Institute of Medical Sciences, New Delhi, India, a tertiary care research hospital. 

## Materials and Methods

### Study Participants and Sample Collection

For our prospective study, we recruited patients with an AFI (history of fever, i.e., temperature >38°C that had persisted for >2 days without an identified source) from the Department of Medicine at All India Institute of Medical Sciences from July 2017 through September 2018. Every admitted consenting AFI patient was tested by PCR for all 5 *Plasmodium* species (*P*. *falciparum*, *P. vivax, P. malaria*, *P. ovale*, and *P. knowlesi*), DENV, CHIKV, *O. tsutsugamushi,* and *Leptospira*. The study was approved by the institute research ethics committee (reference no. IEC-55/07.10.2016, RP7/2017).

For each participant, we collected information about geographic location (Figure) and completed a standard questionnaire (including demographic information, history, general physical examination findings, systemic examination findings, and clinical investigation findings). To determine presumptive clinical diagnoses and treatments, we reviewed medical chart records corresponding to each participant. 

**Figure:**
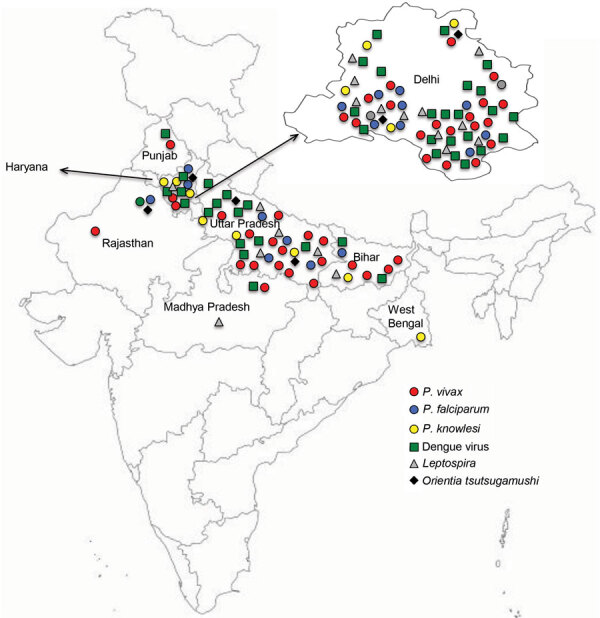
Locations of malaria patients with co-infections, India, July 2017–September 2018. Close-up view of Delhi state is provided.

 All patient data were anonymized to protect confidentiality. Blood samples were collected and subjected to microscopy, rapid diagnostic testing, and PCR analysis for all 5 pathogens (5 species of *Plasmodium*, DENV, CHIKV, *Leptospira,* and *Orientia*). Typhoid testing was not conducted for patients with no abdominal pain or diarrhea. None of the patients recruited for this study showed indications for typhoid testing.

For microscopic examinations, we used peripheral blood smears (Giemsa-stained thick and thin smears) and a 3-band rapid diagnostic test kit (SD Bioline Malaria Ag Pf/Pan kit; Standard Diagnostics, Inc., https://www.alere.com/en/home.html). The rapid diagnostic test detects antigens specific to histidine-rich protein II from *P. falciparum* and pan-*Plasmodium* lactate dehydrogenase from *P. vivax*, *P. malariae*, or *P. ovale*.

Patients positive for malaria by PCR were classified as having severe malaria according to World Health Organization 2015 guidelines (https://www.who.int/docs/default-source/documents/publications/gmp/guidelines-for-the-treatment-of-malaria-eng.pdf?sfvrsn=a0138b77_2). These guidelines define severe malaria as creatinine level >3 mg/dL, bilirubin level >3 mg/dL, bicarbonate level <15 mmol/L, hemoglobin level <7 g/dL for adults and <5 g/dL for children, parasite count 10%, hypoglycemia <2.2 mM, substantial bleeding, impaired consciousness, shock, prostration (defined as myalgia and arthralgia), multiple convulsions, and pulmonary edema) (*26*). The remaining patients were classified as having mild malaria. 

### DNA Extraction and PCR Analyses

From participating AFI patients, we collected 5 mL of venous blood into EDTA tubes for PCR analysis. We extracted DNA from whole blood by using a QiaAmp DNA Mini Kit (QIAGEN, https://www.qiagen.com) according to the manufacturer’s instructions. To detect DENV and CHIKV, we extracted RNA from TRIzol by using the isopropanol method, and we synthesized complementary DNA from RNA by using a Verso cDNA Synthesis Kit (Thermo Fisher Scientific, https://www.thermofisher.com) according to the manufacturer’s recommendations. We analyzed all samples for the presence of all 5 human *Plasmodium* species, *O. tsutsugamushi, Leptospira,* DENV, and CHIKV. All samples were also subjected to microscopy and rapid diagnostic testing (for PfHRP2 and PvLDH genes) for malaria diagnosis. The diagnosis of DENV and its serotypes was conducted by using serotype-specific PCR primers. The presence of other infectious agents, such as *O.* *tsutsugamushi*, *Leptospira*, and CHIKV was detected by PCR (Appendix Table 1). Randomly selected representative PCR products were subjected to Sanger sequencing to confirm species identity (Appendix Table 2).

We categorized the types of infections or combination of infections in a person as monoinfections, mixed infections, or co-infections. Monoinfections are defined as infections with 1 species of *Plasmodium,* mixed infections with >1 *Plasmodium* species, and co-infections with *Plasmodium* species and other bacterial or viral infections.

### Determination of Patient Locations and Construction of Map of India

We were able to retrieve location data for 82 patients. We constructed a map of India based on the official maps provided by the Survey of India (http://www.surveyofindia.gov.in/pages/display/235-political-map-of-india), as described previously (*26*). In brief, we downloaded an India map shapefile (http://www.indianremotesensing.com/2017/01/Download-India-shapefile-with-kashmir.html) and generated the final image by using Microsoft PowerPoint (https://www.microsoft.com) to map each patient to their local area. In addition, the 12 patients with *P. knowlesi* infection were asked to answer questions about time of malaria infection (as recorded in our dataset), travel outside India in 2 years preceding the malaria infection, visits from abroad by friends/relatives, and any previous malaria infections (possibility of recurrence/relapse).

### Statistical Analyses

We recorded data on a predesigned form and managed the data in a Microsoft Excel spreadsheet and checked all entries for possible manual errors. We summarized categorical variables by frequency (%) and age as means. We used χ^2^ or Fisher exact tests, or both, as appropriate, to compare frequencies between 2 groups and the Student *t*-test to compare age distribution between 2 groups. We evaluated accuracy of microscopy and rapid diagnostic testing methods by using PCR as a reference for malaria diagnosis. For each of the 2 tests, we computed sensitivity, specificity, positive predictive value, negative predictive value, positive likelihood ratio, and negative likelihood ratio by using PCR as a reference. We also computed 95% CIs for each measure computed to determine the strength of association of various co-infections with malaria severity. We used bivariate and multivariate logistic regression methods to determine the odds ratio (95% CI) for each co-infection by using Stata version 15.0 statistical software (https://www.stata.com). We considered p<0.05 to be statistically significant. We created a patient baseline characteristics table by using the R version 3.4.3 package tableone (*27*). The tableone package summarizes categorical data in the form of counts and percentages and summarizes continuous data in the form of means and SDs.

## Results

### Spectrum of Co-infections and *Plasmodium* Mixed Species Infections in Patients with Malaria

We analyzed the prevalence of various co-infections and *Plasmodium* mixed-species infections in the 66 *Plasmodium*-positive samples (Table 1). *P. vivax* accounted for most (76%) (50/66) infections, whereas *P. falciparum* accounted for 35% (23/66). *P. knowlesi* was detected in 18% (12/66) of infections (Table 1); *P. malariae* and *P. ovale* were not detected in our study.

**Table 1:** Frequency of co-infections with *Plasmodium* spp. and DENV serotypes 1–4, *Leptopsira* spp., and *Orientia tsutsugamushi*, India, July 2017–September 2018*

Pathogen	No. co-infections
All *Plasmodium-*positive infections, n = 66	
*P. falciparum* alone	10
*P. vivax* alone	34
*P. knowlesi* alone	5
*P. vivax* + *P. knowlesi*	4
*P. falciparum* + *P. vivax*	10
*P. falciparum* + *P. knowlesi*	1
*P. falciparum* + *P. viv*ax + *P. knowlesi*	2
*Plasmodium* + bacteria co-infections, n = 17	
*Plasmodium* + *O. tsutsugamushi*	5
*Plasmodium* + *Leptospira*	11
*Plasmodium* + *Leptospira* + *O. tsutsugamushi*	1
*Plasmodium +* DENV co-infections, n = 40	
*Plasmodium +* DENV, all serotypes	40
*Plasmodium +* DENV-1	8
*Plasmodium +* DENV-3	5
*Plasmodium +* DENV-4	20
*Plasmodium +* DENV-1 + DENV-3	1
*Plasmodium +* DENV-1 + DENV-4	2
*Plasmodium +* DENV-3 + DENV-4	1
*Plasmodium +* DENV-1 + DENV-4 + DENV-3	3
*Plasmodium +* DENV *+* bacteria co-infections, n = 11	
*Plasmodium +* DENV *+ Leptospira*	8
*Plasmodium +* DENV*+ O. tsutsugamushi*	2
*Plasmodium +* DENV + *Leptospira* + *O. tsutsugamushi*	1

*P. malariae and *P. ovale* were not detected in the study population. DENV, dengue virus.

From the 66 *Plasmodium*-positive patients, 40 (60%) samples indicated a DENV co-infection with or without other co-infecting pathogens, and 29 (44%) indicated exclusive *Plasmodium*/DENV co-infections. *Plasmodium* co-infections with bacteria were found for 16 (25%) patients: *Leptospira* infections for 11 (17%) of the 66 and *O*. *tsutsugamushi* for 5 (8%) (Table 1).

Mapping indicated that locations of the malaria patients in our study spanned the entire northern region of India, including the states of Rajasthan, Haryana, Punjab, Delhi, Uttar Pradesh, Bihar, and West Bengal (Figure). Patients with *P. knowlesi* infection originated from Delhi, Haryana, Uttar Pradesh, and West Bengal. Most patients with dengue infections originated from Delhi and Uttar Pradesh. Of the 12 patients with *P. knowlesi* infection, 5 had not traveled abroad or had direct contact with any visitors from abroad for at least 2 years before admission. No information was available for the remaining 7 patients (Appendix Table 3).

### Patient Baseline Characteristics

Detailed hematologic and biochemical parameters for all patients were retrieved from medical records (Table 2; Appendix Table 4). Differences between severe and mild malaria patients were found in hemoglobin levels (9.89 g/dL vs. 12.11 g/dL), hematocrit (29.93% vs. 36.45%), platelet counts (76.69 vs. 87 × 10^3^/μL), leukocyte counts (10.53 vs. 6.07) × 10^3^ cells/μL), and creatinine levels (3.37 vs. 0.90). Each group contained 26 male patients; mean age for severe malaria patients was 28 years and for mild malaria patients was 32 years.

**Table 2:** Comparison of blood parameters for patients with mild or severe malaria, India, July 2017–September 2018*

Parameter	**Mild disease, mean (± SD), n = 33†**	**Severe, mean (± SD), n = 33‡**
**Hemoglobin, g/dL**	12.11 ( ± 3.22)	9.89 (± 2.96)
**Hematocrit, %**	36.45 (± 9.48)	29.93 (± 8.95)
**Platelets, ×10^3^/μL**	87.00 (± 54.73)	76.69 (± 66.24)
**Leukocytes, × 10^3^ cells/μL**	6.07 (± 3.20)	10.53 (± 6.98)
**Erythrocytes, × 10^6^ cells/μL**	4.26 (± 1.34)	3.69 (± 0.95)
**Creatinine, mg/dL**	0.90 (± 0.41)	3.37 (± 3.41)

*In each group, 26 patients were male. Mean (± SD) ages were 32.03 (± 15.99) y for those with mild disease and 28.81 (± 13.99) y for those with severe disease.

### Association of Co-infecting Pathogens with Malaria Severity

We found that co-infection with DENV serotype 4 (DENV-4) was associated with mild malaria (adjusted odds ratio [aOR] 0.3, 95% CI 0.4–5.0), whereas infection with *Leptospira* (aOR 1.6, 95% CI 0.4–6.8) or *O. tsutsugamushi* (aOR 1.1, 95% CI 0.1–7.8) was associated with severe malaria. *P. vivax* or *P. knowlesi* monoinfection was also associated with severe malaria (aOR 2.5, 95% CI 0.9–7.2) (Table 3). Other categories of *Plasmodium* mixed-species infections did not show any strong association with malaria severity (Appendix Table 5). However, the species of *Plasmodium* may confound some of these analyses.

**Table 3:** Frequency of co-infections and mixed infections in patients with severe and mild malaria, India, July 2017–September 2018*

Co-infections	No. malaria cases			Unadjusted OR (95% CI)	Adjusted OR (95% CI)
	Severe, n = 33	Mild, n = 33	p value		
DENV			<0.08		
Neg for DENV	14	12		Referent	Referent
Pos for DENV-4	6	14		0.34 (0.1–1.2)	0.3 (0.4–5.0)
Pos for other DENV serotypes: 1, 3, 1+3, 4+3	13	7		1.6 (0.5–2.5)	1.4 (0.4–4.9)
*Leptospira*			<0.5		
Absent	26	29			
Present	7	4		1.9 (0.5–7.4)	1.6 (0.4–6.8)
*Orientia tsutsugamushi *			<0.5		
Absent	30	31		Referent	Referent
Present	3	2		1.6 (0.2–9.9)	1.1 (0.1–7.8)
Malaria parasite types			<0.1		
*Plasmodium* mixed infections	17	10		Referent	Referent
*P. vivax*/*P. knowlesi* monoinfection	16	23		2.4 (0.9–6.7)	2.5 (0.9–7.2)

*Bivariate and multivariate logistic regression analysis was used to determine the strength of association of various co-infections and mixed malaria infections with malaria severity. DENV, dengue virus.; neg, negative; pos, positive; OR, odds ratio.

### Relative Performance of Malaria Diagnostic Procedures 

All 99 patients were tested for *Plasmodium* species by microscopy (8 positive results), rapid diagnostic testing (26 positive), and PCR (66 positive) (Table 4). Almost 50% of the *P. vivax* infections escaped detection by both microscopy and rapid testing. *P.* *knowlesi* was detectable solely by PCR. In addition, rapid diagnostic testing was able to detect only 1 of 18 *Plasmodium* mixed-species infections (Table 4). The diagnostic performance of microscopy and rapid diagnostic testing was calculated, and each was found to have poor sensitivity compared with PCR (Appendix Table 6).

**Table 4:** Summary of detection of *Plasmodium* species by RDT, microscopy and PCR, India, July 2017–September 2018*

Parasites	RDT, no. (%)	Microscopy, no. (%)	PCR, no. (%)
*Plasmodium* negative	73 (71.7)	90 (90.9)	33 (33.3)
*Plasmodium* positive	26 (28.3)	9 (9.09)	66 (66.7)
* P. falciparum*	6 (6.06)	2 (2.02)	10 (10.1
* P. vivax*	14 (14.1)	7 (7.07)	34 (34.3)
* P. ovale*	0	0	0
* P. malariae*	0	0	0
* P. knowlesi*	0	0	5 (5.05)
Mixed *Plasmodium*	1 (1.01)	0	17 (17.2)
Pan-*Plasmodium*	7 (7.07)	0	0

*Percentages are calculated out of all AFI samples (n = 99). RDT, rapid diagnostic test.

## Discussion

Among patients hospitalized with AFI at the All India Institute of Medical Sciences during July 2017–September 2018, the major circulating *Plasmodium* species was *P. vivax* and malaria/DENV co-infections predominated. A high number of severe malaria cases reported to the institute are from northern India. Among the 5 *Plasmodium* species known to infect humans, in our study population we detected *P. falciparum*, *P. vivax*, and *P. knowlesi* but found no evidence of *P. malariae* or *P. ovale*. Most AFI patients in this study originated from northern India across the states of Rajasthan, Haryana, Punjab, Delhi, Uttar Pradesh, Bihar, and West Bengal. The burden of co-infecting pathogens in patients with malaria was revealed by a combination of complete blood work (peripheral blood smear analysis, rapid diagnostic testing, serum renal and liver function testing) and in-depth molecular assays (PCR amplification of *Plasmodium* species–specific genes followed by Sanger sequencing). We found a very high percentage of *Plasmodium*/DENV co-infections in our study population. This finding can be partly attributed to the highly sensitive PCR diagnostic methods used.

A recent meta-analysis of the prevalence of DENV/ *Plasmodium*/CHIKV co-infections spanning 7 geographic regions (southern Asia, Africa, Southeast Asia, South America, North America, the Caribbean, and the Middle East) showed that DENV/*Plasmodium* co-infections have been reported in 19 countries, including India; DENV/CHIKV co-infections have been reported in 24 countries including India; CHIKV/*Plasmodium* co-infections have been reported in 6 countries with only a single co-infection reported from India; and DENV/CHIKV/*Plasmodium* co-infections have been reported in 3 countries (*5*). According to that meta-analysis, the average reported prevalence of DENV/*Plasmodium* co-infection in India is ≈6.5%, which is much lower than that detected by our study. However, a more detailed analysis from the eastern India state of Odisha shows that this percentage can vary within a year, depending on season, and the highest reported prevalence of DENV/*Plasmodium* co-infections from this region was 31.8% during September–October, an observation similar to ours (*28*).

Although awareness of *Plasmodium*/DENV co-infections is increasing, little information is available about *Plasmodium*/*Leptospira* or *Plasmodium*/*O. tsutsugamushi* co-infections (*13*,*29*). This lack of information is concerning because our study suggests that *Plasmodium*/*Leptospira* co-infections are associated with severe malaria. Prevalence data for co-infections with these pathogens are limited. We emphasize the need for such information because although these pathogens are carried by different vectors, they co-exist in the same geoclimactic habitats that combine a warm, moist environment with dense vegetation and poor socio-economic development (*13*,*29*). The presence of one co-infecting pathogen can influence disease outcomes, treatment outcomes, development of immunity, or drug resistance with regard to infections caused by the other co-infecting pathogen. One example is the predisposition for bacteremia to develop in persons with malaria (*30*).

In most malaria-endemic settings, malaria is still diagnosed by microscopic examination of Giemsa-stained peripheral blood smears and rapid diagnostic testing for parasite antigen. The rapid test is specifically designed to detect *P. falciparum* and *P. vivax* and is extensively used because of its speed and simplicity. For microscopy, diagnostic success depends on the skill of the technicians who observe the peripheral blood smears. We found that rapid tests and microscopy missed most of the *P. vivax*–positive malaria cases and all *P. knowlesi* cases and detected only 1 of 18 *Plasmodium* mixed-species infections. This finding clearly shows the limitations of rapid testing and microscopy for comprehensive detection of malaria parasites, which have been independently observed in several studies and attributed to deletions in the HRP2 and HRP3 genes in the specific case of *P. falciparum* infection (*31*–*34*). This problem is well recognized for healthcare workers and researchers working toward malaria elimination all over the world. Although the rapid diagnostic test for malaria has been shown to be better than microscopic examination of Giemsa-stained peripheral blood smears, PCR has been shown to be far superior to rapid testing for diagnosing low-parasitemia malaria infections (*35*). Our observations were similar; PCR was most sensitive, followed by rapid testing and then microscopy. However, rapid tests have low success rates in areas of low transmission intensities and give rise to a high proportion of false negatives (*36*). In addition, rapid tests fail to detect infections with emerging pathogens, such as the simian parasite species *P. knowlesi* and *P. cynomolgi*, both known to infect humans (*37*). Although recent reports highlight the improvement of rapid tests for *P. knowlesi* detection by use of a cross-reacting pan-parasite lactate dehydrogenase feature, we were unable to detect *P. knowlesi* by using a pan-parasite lactate dehydrogenase–containing rapid test, possibly because of low parasitemia, below the detection limit of the rapid test (*38*). *P. knowlesi*, which was previously believed to be localized to Southeast Asia, has now been reported from various parts of the world as single case reports of travelers’ infections from areas including Oceania, Europe, and the Middle East (*39*–*41*). From India, *P. knowlesi* infection has been reported from the Andaman and Nicobar Islands in the context of drug resistance and in a recent study by our group in the context of acute kidney injury (*26*,*42*). Historically, *P. knowlesi* infection was discovered as a naturally occurring human infection in Malaysia in 1965 (*43*). The accurate diagnosis of *P. knowlesi* by use of PCR took ≈40 years from its initial discovery and gave a preliminary indication of the burden of this zoonosis in Sarawak, Malaysia (*44*). Until this point, *P. knowlesi* as a human infection was frequently misdiagnosed as *P. vivax*, *P. malariae*, or *P. falciparum* infection. 

To assess whether the infections originated locally, we surveyed the *P. knowlesi* patients in our study group for the possibility of travel abroad or interaction with visitors from abroad within their family. The patients who responded to our questionnaire do not appear to have traveled abroad or to have had direct contact with anyone visiting them from abroad, suggesting local presence of *P. knowlesi*. However, unknown sources of travel from Southeast Asia, a neighbor to India, cannot be ruled out. Currently, testing for *P. knowlesi* is not included in diagnostic procedures in India, irrespective of diagnostic method (microscopy, rapid diagnostic test, or PCR), because it has not been widely reported. However, India is known to harbor both the potential vector for *P. knowlesi*, the *Anopheles dirus* mosquito, as well as the reservoir, pig-tailed macaques (*Macaca nemestrina*), thereby making India a potential ecosystem for the proliferation of this zoonotic *Plasmodium* species (*45*). Furthermore, the populations of *Macaca mulatta* macaques and related species have recently expanded in northern India, particularly in the state of Uttar Pradesh, which may explain the appearance of *P. knowlesi* in our study population representative of these areas, whereas it was not reported earlier (*45*). A recent report has also demonstrated the presence of *P. falciparum* parasites in monkey populations from India, indicating freely occurring undetected zoonotic transfer of the malaria parasites across reservoirs and hosts. Therefore, healthcare workers and national programs should incorporate all species of malaria parasites known to infect humans, in their diagnostic portfolio.

In conclusion, our study clearly showed that microscopy and rapid diagnostic testing gave false-negative results for most mixed-species infections and completely missed *P. knowlesi* infections, co-infections and mixed *Plasmodium* infections were highly prevalent in patients with malaria, *Plasmodium*/DENV co-infections were the most common co-occurring pathogens in our study population, *P. knowlesi* infections were present in India, *Plasmodium*/DENV4 co-infections were associated with mild malaria, and *Plasmodium*/*Leptospira* infections were associated with severe malaria. Although the ORs support the above findings, the 95% CIs for these associations were wide. CIs reflect the uncertainty of the estimated effect, and wider intervals suggest greater uncertainty. The wide 95% CIs in this study suggest that although trends were observed, additional data points are needed to determine the effect size of these associations. Wider prevalence studies investigating malaria co-infections are needed.

The government of India has recently declared a goal of malaria elimination by 2030, which will be a major step toward global malaria eradication because India serves as a major *Plasmodium* reservoir, contributing to almost 4% of malaria-related deaths globally. Therefore, to make malaria elimination possible, we offer 2 recommendations based on our observations in this study, particularly for tertiary healthcare centers or centers where the burden of severe malaria cases is high. First, malaria elimination efforts will need to include strategies for malaria elimination in humans as well as animal reservoirs. Second, efforts toward the development of novel diagnostics for malaria must be renewed, and AFI diagnoses must include screening for all 5 *Plasmodium* species, *Leptospira*, and all 4 DENV serotypes.

Appendix Supplementary methods and results for study of association of dengue virus and *leptospira* co-infections with malaria severity.
