# Supplementary material for: Association of Dengue Virus and Leptospira Co-Infections with Malaria Severity
Source: Emerg Infect Dis. 2020 Aug;26(8):1645–53. doi: 10.3201/eid2608.191214 (PMC7392441; doi:10.3201/eid2608.191214)
Supplement: Appendix — Supplementary methods and results for study of association of dengue virus and leptospira co-infections with malaria severity. [file 19-1214-Techapp-s1.pdf]

Article DOI: https://doi.org/10.3201/eid2608.191214

Association of Dengue Virus and *Leptospira* Co-Infections with Malaria Severity

Appendix

Appendix Table 1. PCR primers for detection of *Plasmodium*, Chikungunya, Dengue, *Leptospira* and Scrub typhus species for AFI and AKI samples (*1,2*)*

Plasmodium Species Name	Primer name	Primer Sequence	Primer Length (bp)	Amplicon length (bp)
Plasmodium Primers	Pf-FP[1]	5’-TTAAACTGGTTTGGGAAAACCAAATATATT-3’	30	205
	Pf-RP[1]	5’-ACACAATGAACTCAATCATGACTACCCGTC-3’	30	
	Pv-FP[2]	5’-CGCTTCTAGCTTAATCCACATAACTGATAC-3’	30	120
	Pv-RP[2]	5’-ACTTCCAAGCCGAAGCAAAGAAAGTCCTTA-3’	30	
	Pm-FP[2]	5’-ATAACATAGTTGTACGTTAAGAATAACCGC-3’	30	144
	Pm-RP[2]	5’-AAAATTCCCATGCATAAAAAATTATACAAA-3’	30	
	Po-FP[2]	5’-ATCTCTTTTGCTATTTTTTAGTATTGGAGA-3’	30	800
	Po-RP[2]	5’-GGAAAAGGACACATTAATTGTATCCTAGTG-3’	30	
	Pk-FP[2]	5’-CAGAGATCCGTTCTCATGATTTCCATGG-3’	28	209
	Pk-RP[2]	5’-CTRAACACCTCATGTCGTGGTAG-3’	23	
Chikungunya Virus	CHIK_FP	5’-GAGGCTGCTTTCGGAGAGATTT-3’	22	134
	CHIK_RP	5’-CGGCTGGCGATGGTGATATTTA -3’	22	
Dengue Virus Subtype 1†	DENV1_FP	5’-CCATAGTCCGTGAGGCCATAAA-3’	22	197
	DENV1_RP	5’-AGACAGGAGACGCATAGTGAAAG-3’	23	
Dengue Virus Subtype 2†	DENV2_FP	5’-GTGACTGAGGACTGCGGAAATAG-3’	23	127
	DENV2_RP	5’-ACCCATCCTCACCTCTGTATCT-3’	22	
Dengue Virus Subtype 3†	DENV3_FP	5’-GTTTGGAACAGGGTGTGGATAGA-3’	23	138
	DENV3_RP	5’-TGCTCTGGAAGTGAGACCAATAAG-3’	24	
Dengue Virus Subtype 4†	DENV4_FP	5’-AGATGCCTCAAGCCAGTTATCC-3’	22	122
	DENV4_RP	5’-TGTGCTGGGTTCCTTCCTATTC-3’	22	
Orientia	ST_FP1	5’-TCAAGCTTATTGCTAGTGCAATGTCTGC-3’	28	483
	ST_RP1	5’-AGG GATCCCTGCTGCTGTGCTTGCTGCG-3’	28	
	ST_FP2	5’-GATCAAGCTTCCTCAGCCTACTATAATGCC-3’	30	
	ST-RP2	5’-CTAGGGATCCCGACAGATGCACTATTAGGC-3’	30	
Leptospira	Lau01	5’-ACTCTTTGCAAGCATTACCGC-3’	21	660
	Lau02	5’-AGCAGACCAACAGATGCAACG-3’	21	

***Pf= *Plasmodium falciparum*, Pv = *Plasmodium vivax*, Pm = *Plasmodium malariae*, Po = *Plasmodium ovale*, Pk = *Plasmodium knowlesi*, CHIK = Chikunguniya, DENV= dengue Virus, ST= Scrub typhus, Lau=Leptospira specific primer. FP = Forward primer, RP = Reverse Primer).
†Synthesised in IDT primer synthesis tool.

Appendix Table 2. BLAST results of Sanger sequenced PCR products of *Plasmodium knowlesi* and *Leptospira*

Sample Id	Sequence	Sequence Id	E value	Identity
Sequences for *Plasmodium knowlesi*				
AIIMSK0447_PK_F:	CCCCCAAGACCACGCGGGGGCAACGAATGTAACGTGTTAG<br>GAAGAAAACACCTCAGGATGTGCCAGCATCTTTTGACTTTTA<br>TTTTGGGGGGCTGCATCTACTACCACGACATGAGGTGTTCA<br>GAGATCCGGTCTCATGATTTCGGTGTCCGTGATG	LT727658	2.9	100%
AIIMSK0447_PK_R	ACCTTAAGGAACCAGAGGAGGAGGAACAGCAGAAGAGGGA<br>ATTTCCAGCTACTAACAATTGGTGTAGTTCATCATCACCTGTA<br>ATGACCAAAATGTCTCCCATGGAAATCATGACAACGGATCTT<br>TGATTTTCAAA	LT727658.1	2e-04	93%
AIIMSK1056_PK_F	CCCCCCAGGGTTGTTGTATGAAACAGAACTCTCATATTGTGA<br>GTCGGATGAAAACAACCTCAGGAATGTGCCAGCATCTTTTGA<br>CTTTTATTTTGGGGGGCTGCATCTACTACCACGACATGAGGT<br>GTTCAGAGACCGG	LT727652.1	4e-06	97%
AIIMSK1056_PK_R	CGGGAGAATAGCAAAGCCACAGTTGGATGAGGGTCTTCTCA<br>GGACTAACAATTGGTGTAGTTCATCATCACCTGGAATGACCA<br>AAATGTCTCCCATGGAAATCATCACCCCGGATCTCGGATTTT<br>CAAAT	LT727659.1	0.030	93%
AIIMSK3873_PK_F.	CACTCGACGGTGCAAGGGGGGGCGAAGAGAAAACGTAGTG<br>ATGAGAATCCCTCGGGTTGTGCCAGCATCTTTTGACTTTTAT<br>TTTGGGGGGCTGCATCTACTACCACGACATGAGGTGTTTAG<br>AGATCCGGACTCATGATTTCGATGGTTATCATGACAGCTCTT<br>CTGCTGA	LT727653.1	6e-05	100%
AIIMSK3873_PK_R	CTTACCAAACCGGGGGGACCGGAGGAGGAGGAACAGAAGA<br>AGAGGGACTTTTCAGCCACTAACAATTGGTGTAGTTCATCAT<br>CACCTGGAATGACCAAAATGTCTCCCATGGAAATCATCACCC<br>CGGATCTCTGATTTTCAAA	LT727655.1	0.003	100%
AIIMSK4424_PK_F	TGTTGGGGTAAAGAAGGAACCTCCAGCAATTTGTTATGTGG<br>CTGAGAAGAACCTCAGGAATGTGCCAGCATCTTTTGACTTTT<br>ATTTTGGGGGGCTGCATCTACTACCACGACATGAGGTGTTC<br>AGAGATCCGTTCTCAGGATCCGATGTTGG	LT727652.1	1e-05	100%
AIIMSK4424_PK_R	CTTTGAAAAACATTCCTAGAGGTTCTTTTAAGCCACTAACAAT<br>TTGGGGTAGTTCATCATCACCTGGAATGACCAAAATGTCTCC<br>CATGGAAATCATGAGAACGGATCTCTGACCTCCAAAT	LT727658.1	4e-07	100%
AIIMSK6691_PK_F	CCTTCGAGGGGGGCGCGGCCGGGGGCGAAATGAGAACAGT<br>GAGGCGGTAGAGAGAACCTCAGGAATGTGCCAGCATCTTTT<br>GACTTTTATTTTGGGGGGCTGCATCTACTACCACGACATGAG<br>GTGTTTAGAGATCCGTTCTCATGATTTCGATGGTAGTGATGC<br>GATCCCTTCTGTGA	LT727658.1	5e-06	94%
AIIMSK6691_PK_R	CTAAAGAGGGGGAGACGGCGGCGCGGGCCTGCCGATGCG<br>GGAATTCTCAACCACAACAATCGTCGTTTTTCTTCATCACCT<br>GTAATGACCAAAATGTCTCCCATGGAAATCATCAGACCGGAT<br>CTCTGACCTTCAAA	LT727658.1	2e-04	93%
AIIMSK7167_PK_F	TCAAAGAGCAGAGGAGAGCATAACGATGGTTAAGACTACAC<br>AGATTGTGAGTGGCTGAGAAGAACCTCAGGAATGTGCCAGC<br>ATCTTTTGACTTTTATTTTGGGGGGCTGCATCTACTACCATG<br>GATCTCATGAGAACGGATCTCTGA	LT727658.1	5e-05	93%
AIIMSK7167_PK_R	CTCACTATTATCGGCTCACGAGGGATTGAATCTTTGGACACT<br>GATTCCCAGA	LT727661.1	4.3	100%
AIIMSK8299_PK_F	CTGTCGTAATAAATAGAAGAACAGAGACAAATGAGCCGGAT<br>GAAAAGAATTCAGGAATGTGCCAGCATCTTTTGACTTTTATTT<br>TGGGGGGCTGCATCTACTACCACGACATGAGGTGTTCAGAG<br>ATCCGTTCTCATGATTTCCATGCAACTCATGAGATCTCATCT<br>CCGATG	LT727658.1	1e-07	100%
AIIMSK8299_PK_R	ATTTAGGGAAACATTCGTAGAGGGTTTTCTCAGCCACTAACA<br>ATTGGTGTAGTTCATCATCACCTGGAATGACCAAAATGTCTC<br>CCATGGAAATCATGAGAACGGATCTCTGATTTTCAAAT	LT727658.1	4e-07	100%
AIIMS2045_PK_F	CCCATGCACAGGGGCTCGACCAGTCATGTGGATAAATCACT<br>CTCCTATCGTCGGTGGCCG	LT727655.1	1.1	100%
AIIMS2045_PK_R	GGGGCAAGAAAAAAGGAGGCTTAATACGCTCGGCACGTCGT<br>AGAGGTTCT<br>TCTCAGCCA	LT727659.1	0.036	92%
AIIMS5281_PK_F	TGTTTTTAATGGAGTATGAGGTCAAAAAAGGAGATAAGGGAC<br>CAGAAATTTCCTTTTGTTTTGTTCCTCCCTCTATCTTTTTTAG<br>GGAGGGGGGTTGCAAATTATTTTTTTTTTTTTCGTGCCAGAG<br>ATCCTGTTTTTGATTTCCTTG	LT727655.1	0.089	92%

Sample Id	Sequence	Sequence Id	E value	Identity
AIIMS5281_PK_R	TAGGAAGGATGTCAGGAGGAGAGGGAAGCAGGGGGGAGG<br>CGCGGGACAACTTAGGGTGTATTTCTTCTTCGCCTGGAATG<br>ACCAAAATGTCTCCCATGGAAATCATAATAACTTATCTCTAAA<br>CACTTCATTTCATAATACTTTTCCCCAATTTATTATATAAAA	LT727660.1	0.14	89%
AIIMS7488_PK_F	AGCTGTTGTGGAGGAATGAGAAGAGAGGGAAGGGGGCGAG<br>GAGAAGAGTTTTTTTTTTTTTTTTCATCTTTTGACTTTGGGGT<br>GGGGGGGCTGCTTCTTTACCACTACTTTTTTGTGTTCAGAGA<br>TTCGTTCTCATGATTTCCATGGCAAA	LT727658.1	5e-07	94%
AIIMS2017_7488_PK_R	AGAACCGGATACGCTACGGATGTTGGGAGAGGGGCGGCGC<br>GGCCAGAAGGTTGGTGTAGTTCATCGTCGCCTGGAAGGACC<br>AAAAAGTCTCCCATGGAAATCATGAGAACGGATCTCTAAACA<br>CCTCATGTCGTGGTAGTTTCTTCAAAATTAAAAAC	LT727658.1	3e-05	97%
Sequences for *Leptospira*				
AIIMS3002_M18L_Lau01	TGGAAAAAAACATGGAAAGGACGCAAGCAAAGAAGATCTTTT<br>TTGTGTTTTTTGTTTTTTTTGGTGTTTCTATCTTTTTATGTTTTT<br>TATAGAGAGATTAGAAATGGGAGGGGGGTGTGGGGAAAGA<br>GAGGGAGGCACCAGGGGTGGAGGGGGCCGGGGGGGGGG<br>AGGGGGGGGGAGGAGGAAAAAGAAGAGAAAGTGGGGGAG<br>GGGGGGGGAAGGGGGCGGCGAGGGGCGGGCGGGAGACA<br>GGAGAAAACACGAGGGAGAGAGCGAGACGAGAGCGGGGAA<br>GAGATGGAAGAGGGACGG<br>GGAGGGGGGGGGGGGGAGAGGCGGCGGGGGGGAAAGA<br>AAGCAAACCACCCGCACCACGAAAACCTGGGCGGCAGCCT<br>GGTGGAAAGTGAGCTCAGCTCAGCAGTCCGGAGGGGGGGC<br>ACCGCTTTGAGGTGGGGAGGAAGCCGGGGCTAACGGTGGC<br>AGCAGCTAAGTGAGGACAGGGCGGTGCGATTAACATCTTTT<br>TCAAAGAAGGAAACGCACGAGGCCAGAAGCAGAATCCGCCA<br>AGAGCCCCAAGCAAACAACATAACCTTAAGCGGCAGAAGGA<br>CGATCCCCCTTCCCCCACT<br>AGCTTTAACCAGCAATCGGCAGGAGCAAAAGACCTCCGCGC<br>AGGCGGGGGAGATTCTCATTAGCTAGCGCATCACAAAAAAA<br>ACAAAAAAAAAAAAAAAAATAACAGCCCACAGGCGTACACCA	CP000786.1	0.59	96%
AIIMS3002_M18L_Lau02	TTTTGGTCATGAAGGGCGACAGGCGACGAGCGCGACAGGC<br>GAGACCCATAAAATTTCCCAAATATTATCTTATTATTTTAAATT<br>ATTTTTAATTTTTAACAACTATTTTATTATTAATTAAAATTATTT<br>AAAAATTTTTAAAAATAAAAAAAAATATCAAAGTCTAATTTGTT<br>CTTTTGATCTTCTTTCTTTAACTTCTTTTGTTTGCTTTTTCTTTT<br>GCTTTTTTTTTTTTTTTTTTTTTTTTATTTTAATTATTTTTGTTTTG<br>CGTATGATTGGGTGTGGACTTAGGATTAAGTGCGGTTGGGT<br>TGAAGGGTGGAGCTCGAGATGCAATTAGTCAGTGAAACGGC<br>CGGAATAATATGGCCATGCCTCATAGCGGGTCAGGACCCAT<br>GCGCTCTCAATACCGCCGAGGCCGTCCGTTAGACTTAAGCG<br>CAAAGCCCGGGGTGCGGAAGGAGTCAATCACTCGATGAAG<br>GCGAGGAGCCCGCTGTCCGCTGCCATAAGGGA<br>ACAGCGGGCCAGTGCAAGGCGAACGGCAAACGAGGGGATA<br>CATGGGCTGAATGGCGGACGTAAACGAAGCGCGGACGTGA<br>GTGGTACGCTGGGCGTACGAGAGGGGAGCGGATCACGCGT<br>CGAAACGAAACACATTATCACCAATCGTGG<br>AGACGCATGTCCACATAGACGCGGATCTCAGCGCACAACAT<br>CGGTCGTCGGTGCTGGGCAACAGGGTCGCACCAGAGACAC<br>GCATATGACGTCACTGGCGATGACGCACAGACACATTGCGT<br>GCTGGCGATGAAGACTCGGCGCTGGGGCTATCGGTAGGGG<br>AGCTCCCTCTACTCGGA	CP020414.2	8.1	76%
AIIMS9124_M18L_Lau01.(FP)	TTTTTTTGGTTGGTGGAGCAGAAAAGAAGCAAAAAAAGCCG<br>GAAGGAAATCTCTCTTGGTTGTCTTTTTTTGTCTGTTCGCTCA<br>CTTGTGCCATTTGGTCTGCATGTCTGCCTTCTGTTTCGTGAC<br>TTTCTTTAGTTTATCTGTTGCCATGTTAGGAGGGGAGCGGGG<br>GGGGTGATGGCTGGTTTATTAAGGTGGGCTTAATGGGGGCG<br>AAGAGGGGCCCCCGGTGGGGAGGAAGGAGGTTTGCGCCG<br>CCACTATGGCGGGCTGGAGAATTTGGAAGAGAGCGGGGGG<br>AAAATTAAACCACGAAATTTCAATGAGGGGAGGTGAGGGGA<br>CTGAAAATAATTTTACCTTTGGATCTTCAAGCTCGGGGTGGG<br>TGCGATCCCCACCTAAAGCTGACGGAAGGGGAACCCCCAA<br>GGGGTGGTTTGCATCCACCCGACAGAGGATAAGGGAGCGG<br>GAAAACAGGGAAGGGTTGCTCGTACGCCCCCGGTCCCTTG<br>GTGGGGGGGCGAACCAAAAAACCAACCAGACTCCCTCCCTT<br>CACAAAAACCGAAACCGAAATAAGAAACAAAAATGAACCGAA<br>TCCCCCTCACCTCTATTTGTAGCTTGATTACCACATACATAAT<br>TGAATCCCACCGGAAACTGTGTGGGAGCCAGAATCCGCGCA	CP015217.1	0.77	89%

Sample Id	Sequence	Sequence Id	E value	Identity
	ACAGGTACTTTCGTTCTTGCTACACGCACTACTGCACTTGACACTTAAAACTATAGAGAATCGCCACCTCCGACCCCTACTTATAATATTGGTATGCATCGACTAGCTCCCTCGTTGTGCAGACAGCACATGAGACTGATCAGCACTGCCGCTGACGTAGCGTGTCTGTTGTAGCTGCGCCGGAGAGAGAATCAGACGCGTCTCATGGGCGGTGCGCGTGTGCGGTGCGCGCGGACCCACCTCTGATCGACACCTAATGCAACGAGATATATGCGCTTACAGAGATGCACGCAGAGATCATGTGAGCGGGCAAAACGCCCGTAGTGCATACGAAAAGATGAGTAGATATCTGCTCTCCGTCCTGCATGCCTTCGTCGACGTCGATCGGGGCGGCAAGTGATCAGTCGCAGGAGGCGTACTGCCTGCCGCGCGCCGGGAGTCACGGAATACGTGAGTACGGCGGTAGATGGGACGTAAGTACGCTGCTAGTCTGACTGGTCCCTGCAGTCAATAAAACCCTCGT			
AIIMS9124_M18L_Lau02	TATGCCTTGTTAGGGAGCGGCGTGCGACGAGCGAAGAGGGAAGCGACACACATAGTTCTCAATTATCTTTTATGTACTTTTGCTACTATTCACGAGTTACTTCTTACACCTCTGCTTTCCATTTCCATTTTTACATTATTTTATGCTCTGTTTGCTTTCCATCTCTCTCACCCGCGATTCTTAGACGACGTGGCAGGAGCTTGTGGGGGCTGGAGCATATATGTTCAGGGGGAGGGACATATATCGGTGAGGAAGAATGGGTAGCCTGCCAGGAGCGATTGGGAGTGGCGGCGGGGGCGGCGGCTTAAATCACTGCCGAGACGGCAGGAGCGTGAACAAAAGGTGCCACCCGTGATTTAAGGGGCAGGGGCGTAAATAAGCCGAATCCAGATTCAGAATAACACGGCCCATGTTAGGACCGCTGTTTCGTGAGCTTGAAACCGGCGGTGGAAGAATACCCAGAGAGGGAAAGGACTTAGATAGAGCGCGACTGGTGGAACAAAGGACTGGGATAGAACAGGCCAAGTCCGTCGGAGGCGAAATGAGGGGAGAGCCGACCGGGGGAAGAATGGGCCATGAGAGTTAGGGAGCACAACAGGAGAAAGCGCGGTGGACGAGGGGGGGGCGTTAAAAGTGGGAAGGTTCCATAAGATGAGTTAAAGGTCGAAAGTTACGCCGGGATCCCCGACCTGGACGAGAAGGCAGCGGTGGTGGAGGTACAATCCCTCAAGTCTACCGAAGCAGATAGCAGTGAACAGGGGCGGGCGCTTTGGGGTCGGCAAACTGGATGGGACCTTGGTGGACCGATCTGGATATTTTGTCAGCTGGAGGGTGGTGCAAGTCATATATAGTGGAGGTTGTGGGGGTTGGAGTAATAGTGTTGTGCTTTCGGC	CP030142.1	9.0	100%
AIIMS1071_M17L_Lau01	TTTGAAATAAATGAAGGACAAGGAAGAGGAAGAACAAAAAGAAGAAATGTTTTTTATTTTTCTCCTATTCTTTTTGCGTGCTTATTTCCTGCCAGGTACCATGATACCCCCTTAACATATTACCTCATTTAATTGTCACCACAACCGTGTGAGGTGGTTCCTATTCTCTCCAAGCTACAGTTGAAGAAATGAGCGTCATCATGTCCCTCTTGGTGCACTCTCGTTCGGGAACCAGCTCTTTGCATCCACTCTTAACGGTTCTGGCAGATAATCTTAAATGAGCTGGTGCCTATTAATCATTTACCGAGTTTGATGTGTGGAAGTGTGAGCGGAGAACAATTAAGCTTAGGATCTAGAACTCGAGCTCGGTACCAGGATCTCACCAAAGCCAACTGACAGGGGAAACCCCCTGGATGGAGGTCCTGACTTCTCCCGACCGGAGGAGAATAGCGAGATGGGAAAAAAGGATAGGATGGGCGAAATGCAACGAATCCCCTGGTGGGTGGGGTGAGCGCAAAACCATTAACCCGCCCTATTCTCTCGCTGAGTCGAAATCGAATCACGAGTAGAAAGAGCACTAAATCCGATCGACCTTACAGTGCTGGCTGATTACCACGTATCAGATTGAAAGCCACCTGCACGTACTGAGCGCCAGATCAGCGCACATGGTACATCAGCCTGTCGACTGCGCATTACATGCATGGACGCCAGAAATTATGGATAATCGACTCCTCTATCTAGCTACTTAGATATGGGGGATCTCTGAGCTGCTCGCGCGTTTCGTGATGACGTGCATACTCTGACCATGCAGCTCCTGGAGACGTCACTGCTGCTGTAGCGATGCCGGAGCAGCAGCCGTCAGCCCGTCACGGTGCTGCGGTGTCGGCTCGCATGACCGTCACTGTACGTCTCGAGTGCATCTGCCTAACTATGCGTATCGAGCTATGACTGAAGTGCACCATGCGATGAATACGGTAAGGCTAAGAAGTCCGTACGGACTTTCCGTTCTGCTCATGACTCTGCCTGGCATCCGGTGCGCAAGGTACGCTCTTGAGGCTATCGTTCGGATCGGGATCGTACTGCAGCTACGCACTAGGCGGTGTG	CP000786.1	0.024	93%
AIIMS1071_M17L_Lau02	GTGCCAGACAGGTGGGACAGGAGAGAGAGAGGGGGGGGTGGCGGGAGGGTTGAGTTAGGAGGCAACAACCAGCGCGAAGCTCTTCGGGGGCCTGAGTTTTTACGTACAACCTTGATGCTCTGCATTCCATTTGCAATTTTACATTAATTTATGCAACTGTTTGGTTTCCATCTCTCTCTCCCAAGATTCTTACCACCACGTGGCAG	CP022885.1	4.1	89%

Sample Id	Sequence	Sequence Id	E value	Identity
	GAACATGTGTCAGTTGTAGCCCCACTGTCCTCATGCTGTGT<br>GACACATGGCTCCAGCCTGGGGTGACACAACTGAAAGGGG<br>TGAAATGCTGAATGGCGCAGTTGCCCCTCCATCTTCGTTGAT<br>CCTCAAGGTGTGGAACGGGGAGCGGAATACCCTTAAGCTTA<br>GGATCTTGAATTCGCGCTCGGTACCAGATC<br>ACACCGAAGCTAACGGACAGGAGAAGCCCCACGGATAGAG<br>GTCGAACGTCTCGAGACAGGAGGAGAAAAGCGAGATGGGA<br>AAAAAGGAAAGCATGGGCGAATGCAGCAAATCCCCTGTTGG<br>TTGGGGGAAGCGCAAAACCAGTTAACCGC<br>CCTATTCTCACGCTGAATCGCAAACCGAAATCACGAGTAGAA<br>AGCGCACTAAATCCGATCGACCTTACAGTGCTGGCTGAATA<br>CCACGAACAGATTGAAAGCAACCTGCAACGTATTGAGCGCA<br>AGAATCAGCGCACATGGTACAGCAAGCCTGGCGAACGCGG<br>CATAACATGCAGTGGACGCCAGAAAACTAAGGGAAAATCGA<br>TTCCTCTTATCTAGTTACTTAGATATTGGGGGGATCCTCTAG<br>AGCTGCCTCGCGCGGTTCGGTGATGACGGTGAAAACCACTG<br>ACACATGCAGCTCCCGGAGACGGGCACAGCTTGGCTGGAA<br>GCGGAAGCCGGGAGCAGAGAAGGCCGGCAGGGCGCGCCA<br>GCGGGTTGTGGCGGGTGTCGGGCGCGCCCTGAGACACCAC<br>GTAGCGACGACGGAGTGTAGACTGGCGTAATATGCGGCGT<br>CAGAGCAGATGATGTGAGAAGCGCGATCTGTGGCGTGAGG<br>AACGCCCGTAGGCGGATGGCGAAAAACGCCCAAGGGGGCG<br>TCGCTACCTCCTAACGAATGACTGGCGCGGTCGTTGGTTGG<br>GGGGGGGGAGGGGTCCACCCAAGCGGGGGGGAGTGCGCC<br>CCCCACGGGGGAGGGCGGGGCGGGAAAGACGGGAGGCCA<br>GGGCCGGCAAGGGCGAAAAAGAAAAGGGGCGGGGGTGGG<br>TTGTTCCCAGCCCCGCGCCGGGGAAAAACTAAAAAATGGAC<br>GGGGGGAAGGGGGGAAAATAAAGCAAGAATTAACGGGGGT<br>CCCCCGGAGCCGCTTGGCGGGCCGGGGGACAGGGGGCAT<br>CCATAAGGAGGGGGTTCCCCCGAGGCG			

Appendix Table 3. Travel history of the 12 patients detected to have *P. knowlesi*.

Patient ID	Place of origin	Visiting abroad/within India 2 years before infection	Meeting with relatives returning from Abroad	Recurrence of infection
AIIMS7488_M17	Delhi	Srinagar (Jammu and Kashmir) (India)	No	No
AIIMS2332_M17	Delhi	Not travelled	No	No
AIIMS2045_M17	No data available	No data available	No data available	No data available
AIIMS2045_M17	Delhi	Not traveled	No	No
AIIMS5934_M17	Delhi	Kolkata (West Bengal, India	No	No
AIIMS4184_M17	Delhi	Not travelled	No	No
AIIMS0964_M17	No response	No response	No response	No response
AIIMS4118_M17	No response	No response	No response	No response
AIIMS8310_M17	No response	No response	No response	No response
AIIMS2721_M17	No response	No response	No response	No response
AIIMS6987_M18	No response	No response	No response	No response
AIIMS0694_M18	No response	No response	No response	No response

Appendix Table 4. Whole blood parameters for all malaria positive samples

Parameter	MM		SM	
N	33		33	
Age in y	32.03	(15.99)	28.81	(13.99)
Sex (Males %)	26	(78.8)	26	(78.8)
Hb g/dl	12.11	(3.22)	9.89	(2.96)
HCT%	36.45	(9.48)	29.93	(8.95)
Platelet*10^3/µl	87.00	(54.73)	76.69	(66.24)
WBC*10^3/µl	6.07	(3.20)	10.53	(6.98)
N %	55.58	(15.77)	65.45	(17.92)
L %	31.06	(12.22)	25.17	(14.75)
E %	2.06	(3.31)	1.79	(3.21)
M %	10.54	(4.59)	8.05	(4.53)
B %	0.90	(1.87)	0.72	(0.94)
Prothrombin time (PT)	17.67	(18.09)	16.08	(8.21)
INR	1.66	(1.82)	1.36	(0.39)
Aptt	33.97	(7.81)	32.47	(6.67)
ESR (mm/Hr)	39.17	(22.42)	50.00	(0.00)
RBC *10^6/µl	4.26	(1.34)	3.69	(0.95)
Bil-T (mg%)	1.10	(0.79)	3.31	(5.18)
Bil-D	2.32	(1.63)	2.52	(2.54)
Bil-I	1.21	(2.35)	1.89	(2.54)
AST(SGOT)(U/L)	199.72	(464.03)	193.43	(253.56)
ALT(SGPT) (U/L)	142.97	(332.09)	125.10	(186.63)
ALP (U/l)	335.44	(217.89)	429.43	(249.74)
Total protein(g/dl)	6.39	(0.79)	5.98	(1.05)
Albumin	3.39	(0.62)	3.07	(0.79)
Globulin	2.99	(0.66)	2.94	(0.77)
Urea (mg%)	38.67	(35.28)	93.19	(83.60)
Creatinine (mg%)	0.90	(0.41)	3.37	(3.41)
Na(mEq/L)	139.06	(4.64)	140.34	(5.19)
K	4.35	(0.47)	4.55	(0.71)
Ca (mmol/ltr)	5.57	(3.08)	5.96	(3.62)
Phosphate	4.10	(1.74)	5.25	(2.54)
Cl	110.18	(5.27)	106.67	(23.71)

Appendix Table 5. Distribution of *Plasmodium* species in total *Plasmodium* infection severe malaria (SM) and mild malaria (MM) irrespective of other co-infecting pathogens (DENV, *Leptospira* and Scrub typhus).

Malaria types	Total (n=66)	Severe Malaria (n=33)	Mild Malaria (n=33)
Pf alone	10	5	5
Pv alone	34	20	14
Pk alone	5	2	3
Pf+Pv	10	6	4
Pv+Pk	4	3	1
Pf+Pk	1	1	0
Pf+Pv+Pk	2	2	0
All Pf	23	14	9
All Pv	50	31	19
All Pk	12	8	4

Appendix Table 6. Diagnostic characteristics of Microscopy and RDT methods to detect malaria with PCR as the reference method

	Microscopy(Test)				RDT(Test)			
Diagnostic characteristics	True Positive	True Negative	False Positive	False Negative	True Positive	True Negative	False Positive	False Negative
PCR (Reference)	8	32	1	58	22	4	29	44
%Sensitivity (95% CI)*	12.1 (5.3, 22.5)				33.3 (22.2, 46)			
%Specificity (95% CI) †	97 (84.2, 99.9)				87.9 (71.8, 96.6)			
Positive Predictive Value (95% CI) ‡	88.9 (51.8, 99.7)				84.6 (65.1, 95.6)			
Negative Predictive Value (95% CI) §	35.6 (25.7, 46.3)				39.7 (28.5, 51.9)			
Likelihood Ratio (+)¶	4 (0.5, 30.7)				2.7 (1.0, 7.3)			
Likelihood Ratio (-)#	0.90 (0.8, 1.0)				0.8 (0.6, 0.9)			

*Positive test result among positive by PCR
†Negative test result among negative by PCR.
‡Positive by PCR among positive test result.
§Negative by PCR among negative by the test result.
¶Per false positive by the test, number of true positives detected by the test.
#Per true negative by the test, number of false negatives detected by the test.

References

1. Snounou G, Viriyakosol S, Zhu XP, Jarra W, Pinheiro L, do Rosario VE, et al. High sensitivity of detection of human malaria parasites by the use of nested polymerase chain reaction. Mol Biochem Parasitol. 1993;61:315–20. PubMed https://doi.org/10.1016/0166-6851(93)90077-B

2. Lucchi NW, Poorak M, Oberstaller J, DeBarry J, Srinivasamoorthy G, Goldman I, et al. A new single-step PCR assay for the detection of the zoonotic malaria parasite Plasmodium knowlesi. PLoS One. 2012;7:e31848. PubMed https://doi.org/10.1371/journal.pone.0031848
